# Forty-fifth Annual Report, on the State of the Asylum for the Relief of Persons Deprived of the Use of Their Reason

**Published:** 1862-05

**Authors:** 


Forty-fifth Annual Report, on the state of the Asylum for the relief of persons deprived of the Use of their Reason. Published by request of the Contributors. Third month, 1862. Philadelphia.

This report has reference to one of the oldest and, perhaps, best managed asylums for the Insane in this country.

The present Medical Superintendent and author of the report is Dr. J. H. WORTHINGTON.

